# Genetic toggle switch controlled by bacterial growth rate

**DOI:** 10.1186/s12918-017-0483-4

**Published:** 2017-12-02

**Authors:** Joanna Jaruszewicz-Błońska, Tomasz Lipniacki

**Affiliations:** Institute of Fundamental Technological Research, Polish Academy of Sciences, Pawińskiego 5B, Warsaw, 02-106 Poland

**Keywords:** Mathematical modeling, Stochastic simulations, Regulatory pathways, Bistability, DNA replication, Gene copy number

## Abstract

**Background:**

In favorable conditions bacterial doubling time is less than 20 min, shorter than DNA replication time. In *E. coli* a single round of genome replication lasts about 40 min and it must be accomplished about 20 min before cell division. To achieve such fast growth rates bacteria perform multiple replication rounds simultaneously. As a result, when the division time is as short as 20 min *E. coli* has about 8 copies of origin of replication (*ori*) and the average copy number of the genes situated close to *ori* can be 4 times larger than those near the terminus of replication (*ter*). It implies that shortening of cell cycle may influence dynamics of regulatory pathways involving genes placed at distant loci.

**Results:**

We analyze this effect in a model of a genetic toggle switch, i.e. a system of two mutually repressing genes, one localized in the vicinity of *ori* and the other localized in the vicinity of *ter*. Using a stochastic model that accounts for cell growth and divisions we demonstrate that shortening of the cell cycle can induce switching of the toggle to the state in which expression of the gene placed near *ter* is suppressed. The toggle bistability causes that the ratio of expression of the competing genes changes more than two orders of magnitude for a two-fold change of the doubling time. The increasing stability of the two toggle states enhances system sensitivity but also its reaction time.

**Conclusions:**

By fusing the competing genes with fluorescent tags this mechanism could be tested and employed to create an indicator of the doubling time. By manipulating copy numbers of the competing genes and locus of the gene situated near *ter*, one can obtain equal average expression of both genes for any doubling time *T* between 20 and 120 min. Such a toggle would accurately report departures of the doubling time from *T*.

**Electronic supplementary material:**

The online version of this article (doi:10.1186/s12918-017-0483-4) contains supplementary material, which is available to authorized users.

## Background

Chromosome configuration depends on the nutrients availability. At optimal temperature high availability of nutrients allows *E. coli* to shorten its doubling time to as little as 20 min, while at low nutrients concentration the doubling time can reach several hours. In *E. coli* DNA replication lasts approximately 40 min and must be accomplished 20 min before division [1–3]. As a consequence, shortening of the doubling time to less than 60 min can be only accomplished when replication begins in one of the previous cycles. In this case, the number of origins of replication (*ori*) is larger than the number of the termini (*ter*) for most of the cell cycle (see Additional file 1: Figure S1). Generally, the *ori* to *ter* ratio increases as the doubling time shortens. In *E. coli* this ratio can change from approximately 1 for slowly dividing bacteria to approximately 4 for the shortest doubling time equal 20 min [1]. Consequently, the average copy number of genes closer to *ori* is larger than those near *ter*, and thus shortening of the cell cycle influences expression patterns of genes. Cells exploit this feature by placing the genes involved in transcription and translation in the vicinity of *ori* [4]. Thanks to such genome organization, the number of ribosomes and RNA polymerases increases about 10-fold allowing for fast divisions and simultaneous cell size increase [5]. One can also expect that shortening of the cell cycle will influence the dynamics of regulatory circuits involving genes placed at distant loci. In this study we analyze the influence of the doubling time on the dynamics of a genetic toggle switch in which one of the competing genes is placed in the vicinity of *ori* and the other in the vicinity of *ter*.

A genetic toggle switch is one of the simplest and possibly most studied regulatory elements introducing bistability [6, 7]. Bistable systems, like Lac operon [8, 9], lysis/lysogeny circuit of bacteriophage lambda [10], or competence development in *B. subtilis* [11], allow bacteria to divide into distinct sub-populations. Such systems have been studied both experimentally and theoretically, as well as by means of synthetic biology [12, 13]. In theoretical studies, cell divisions were either accounted implicitly (by protein dilutions) [12–14], or it was assumed that interacting genes replicate at the same time point of the cycle [6, 15–18]. Also in synthetic biology research the competing genes were introduced on the same plasmid [12]. If both genes are replicated simultaneously, the doubling time can influence toggling times [17], but does not shift the balance from one gene to the other.

In this study we explore the case in which competing toggle genes are placed at distant loci. Such a system can be engineered using CRISPR/Cas9 genome editing that allows for inserting designed DNA fragments into desired loci [19]. We show that by manipulating the positions of the genes and number of each gene repeats, it is possible to balance the toggle for an arbitrary doubling time. Then by fluorescent tagging of the competing proteins (as in [12]), one can monitor responses of the system to change in the doubling time.

## Results

**Fig. 1:**
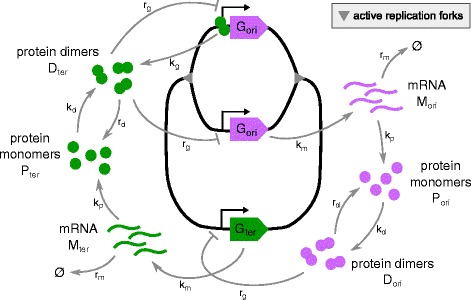
Schematic of the genetic toggle switch in a growing cell. Schematic shows a replicating bacterial chromosome with the toggle genes inserted in the vicinity of *ori* (*G*_*ori*_) and in the vicinity of *ter* (*G*_*ter*_). For simplicity, for both genes only one inserted copy is shown. As the chromosome is shown during the replication process, *G*_*ori*_ is already replicated while *G*_*ter*_ is not yet replicated. Processes of transcription, gene repression and activation occur with the same rates for both genes. To illustrate gene repression and activation processes the two copies of *G*_*ori*_ are shown in different states. The ‘upper’ copy is in a repressed state with a dimer *D*_*ter*_ bound to the promoter. *G*_*ter*_ and the ‘lower’ copy of *G*_*ori*_ are in an active state

**Fig. 2:**
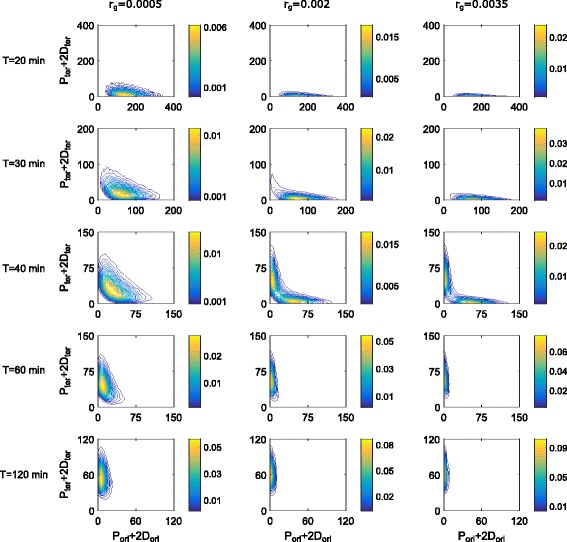
Stationary distribution of protein levels. Stationary distribution of protein levels (total protein content), based on stochastic simulations for different doubling times *T* and different levels of repression *r*_*g*_. The corresponding marginal probability distributions are shown in Additional file 5: Figure S5

We consider a stochastic toggle switch model to analyze the expression of two competing genes in dividing bacteria with respect to the doubling time *T* (see “Methods” section for details). To parametrize the model we use data collected for *E. coli* (Table 1). The competition between the toggle genes arises as each of the two encoded proteins can homodimerize and fully repress the transcription of the opposing gene by binding to its promoter, Fig. 1. Homodimerization provides the additional layer of non-linearity, which together with mutual inhibition of toggle genes, allows for system bistability (see “Model of the genetic toggle switch” subsection in “Methods” section). In the model the strength of the gene competition is regulated by a parameter *r*_*g*_, which describes the repressor – promoter binding rate. When *r*_*g*_=0, the two genes are uncoupled. For a low level of repression, the system (in its deterministic approximation) is monostable, while for large *r*_*g*_, it exhibits bistability, manifested by bimodal protein distributions (Fig. 2).

**Table 1:** Model parameters

Model parametrization	Symbol	Value	Parameter range for *E. coli*
Doubling time	*T*	from 20 to 120 [min]	20 [min] ÷ days
Scaled cell volume just after division (t=0)	*V*_0_(*T*)	$\frac {1}{6}\left (\frac {40}{T}\right)^{2}+\frac {1}{3}$	0.4÷3[*μ* *m* ^3^]^a^
Cell volume	*V*(*t*,*T*)	$V_{0}(T)e^{\ln (2)\frac {t}{T}}$	^b^
Number of gene copies averaged over all genome loci	*S*(*T*)	$\begin {array}{ll} \frac {9T}{80}+\frac {175}{T}-6.5 & T<30\\ \frac {T}{80}+\frac {85}{T}-0.5 & 30\leq T<60\\ 1+\frac {40}{T} & T\geq 60\\ \end {array}$	^c^
Approximate *S*(*T*)	*S*_*appr*_(*T*)	$\frac {1}{\ln (2)}\left (\frac {T}{40}+\frac {1}{2}\right)\left (2^{\frac {40}{T}}-1\right)$	^c^
Gene repression by protein dimer binding	*r*_*g*_(*t*,*T*)	${5,20,35}\times \frac {10^{-4}}{V(t,T)}$	^d^
Gene activation by protein dimer unbinding	*k*_*g*_	2×10^−3^	^d^
mRNA transcription from active gene	*k*_*m*_(*T*)	$\frac {5}{S(T)}\times 10^{-3}$	≤ 0.8[1/*s*]^e^
Protein translation	*k*_*p*_(*T*)	$2V_{0}(T)\frac {40}{T}\times 10^{-2}$	10^−2^÷10[1/*s*]^f^
Dimer formation	*k*_*d*_(*t*,*T*)	$\frac {10^{-3}}{V(t,T)}$	$\begin {array}{c} 1.6\times 10^{-6}\div 9.5 \\ {[}1/(\text {mlcl}\times \textit {s})]\textsuperscript {g}\end {array}$
Dimer dissociation to monomers	*r*_*d*_	10^−1^	$\begin {array}{c} 5\times 10^{-8}\div 1.9\times 10^{3} \\ {[}1/\textit {s}]\textsuperscript {h}\end {array} $
mRNA degradation	*r*_*m*_	3×10^−3^	10^−2^÷6×10^−4^[1/*s*]^i^
Protein monomer degradation	-	0 (only dilution)	$ \begin {array}{c} 1.4\times 10^{-5}\div 10^{-2}\\ {[}1/\textit {s}]\textsuperscript {j}\end {array}$

^a^Cell volume measurements from [30]. Cell size measured as cross-sectional area in range: 2÷7.5[*μ* *m* ^2^] [25, 26]. (Mass/cell) range: 1.3÷5.9[OD_460_ units /10^9^ cells] [5]. *V*_0_ was fitted based on cross-sectional area measurements which are correlated well with cell mass measurements by optical density of the culture [25]

^b^Exponential cell growth based on [30, 31]

^c^Average number of genome equivalents/cell: 1.6÷4 [5]. Average number of *ori* range: 2÷9 [5, 25]. Average number of *ter* range: 1.2÷2.1 [5]

^d^Gene switching is causing mRNA bursts observed at an *E. coli* promoter [32]

^e^For *E. coli* maximal transcription rate: 0.16−0.84/s [33]

^f^Translation initiation intervals are of the order of seconds, although they are specific for each mRNA [34]. In *E. coli* translation initiation rate may vary at least 1000-fold [35]; maximal peptide chain elongation rate: 20*a* *a*/s [36, 37]; average peptide chain elongation rate: 12*a* *a*/s [33]

^g^All cell types: 9.8×10^2^/(*M*×*s*)÷5.7×10^9^/(*M*×*s*) [38]; for 1 *μ* *m* ^3^ volume cell: 1.63×10^−6^/(*mlcl*×*s*)÷9.47/(*mlcl*×*s*)

^h^All cell types: 5×10^−8^/*s*÷1.9×10^3^/s [38]

^i^The vast majority of mRNAs in a bacterial cell are very unstable, with a half-life of about 3 min (decay rate 3×10^−3^/s) – bacterial mRNAs are both rapidly synthesized and rapidly degraded [39]. In *E. coli* mRNA half-lives span between 1 and 18 min (decay rates 10^−2^/*s*÷6×10^−4^/s) [40]

^j^Most of bacterial proteins are very stable, with degradation rates: 1.4×10^−5^÷5.6×10^−5^/s [41]

We assume that one of the two competing genes termed *G*_*ori*_ is placed in the close vicinity of *ori*, while the other termed *G*_*ter*_ is placed in the close vicinity of *ter*, Fig. 1. For most of the analysis we will assume *G*_*ori*_ is inserted to the genome in one copy, while *G*_*ter*_ is inserted in two identical copies. As shown in Additional file 2: Figure S2 the ratio of *ori* to *ter* (averaged over cell cycle) increases as the doubling time decreases. Let us consider two genes placed at distances *L*_1_ and *L*_2_ from *ori*, where the distance is understood as the length of the appropriate DNA segment (in this case shorter segment between gene loci and *ori*) measured in units in which distance between *ori* and *ter* is equal 1. Because the chromosme is circular, two distant genes meay have the same distance from *ori*. It was shown that the average ratio of copies of two genes placed at different distances from *ori* changes with the doubling time and can be approximated by the following formula [20]:

1 $$  R_{1/2}=2^{\frac{C}{T} (L_{2}-L_{1})},  $$

where *C* is the genome replication time, equal to approximately 40 min for fast and moderate growth rates in *E. coli* [1, 2]. Thus *ori* to *ter* ratio *R*_*O*/*T*_=2^*C*/*T*^, as in this case *L*_2_−*L*_1_=1. Let us notice that *R*_*O*/*T*_=2 for *T*=40 min and thus, since *G*_*ori*_ is inserted to the genome in one copy, while *G*_*ter*_ is inserted in two copies, for this doubling time both toggle genes are present in the same averaged number of copies. Additionally, because *C*=*T*, both genes are replicated at the same time and their copy numbers remain equal throughout the whole cell cycle. Therefore, for *T*=40 min the considered toggle is fully symmetric, i.e. it switches between two equally probable states; in each state the expression of one gene is dominant, while the expression of the other is suppressed.

**Fig. 3:**
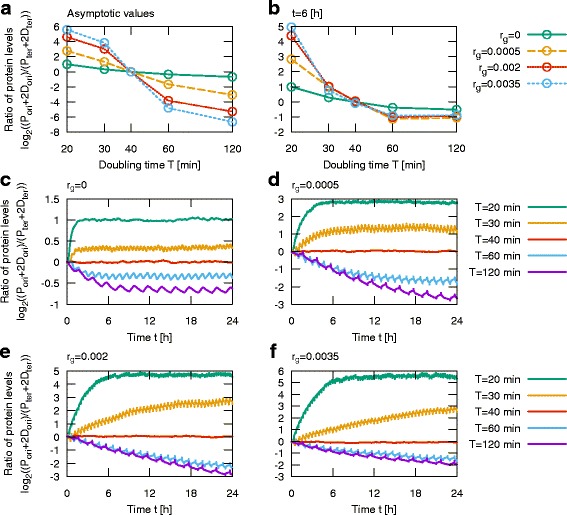
Ratio of population average expression of competing genes for different doubling times *T*. **a** Expression ratio as a function of doubling time *T* in equilibrium. **b** Expression ratio at 6 h after growth rate change from doubling time 40 min to doubling times as given. **c**-**f** Ratio of competing genes expression after growth rate change from doubling time *T*=40 min to doubling times as given. Ratio of protein levels (total protein content) was estimated from 1000 stochastic simulations

For *T*<40 min, *R*_*O*/*T*_>2 and thus gene copy number for *G*_*ori*_ is higher than that of *G*_*ter*_, and consequently the expected expression of *P*_*ori*_ is higher than that of *P*_*ter*_. Analogously, for *T*>40 min, *R*_*O*/*T*_<2, and the expression of *P*_*ori*_ is lower than that of *P*_*ter*_. In the case when repression parameter is low (*r*_*g*_=0.0005), the toggle remains monostable (Additional file 3: Figure S3), protein distribution is unimodal, and gene *G*_*ter*_ gradually prevails over *G*_*ori*_ as *T* increases from 20 to 120 min (Fig. 2). For higher repression parameters (*r*_*g*_=0.002, and *r*_*g*_=0.0035) the toggle exhibits bistability (Additional file 3: Figure S3), and thus it preferentially switches to one of its steady states when *T* becomes longer or shorter than 40 min. The average ratio of competing proteins *R*_*protein*_=(*P*_*ori*_+2*D*_*ori*_)/(*P*_*ter*_+2*D*_*ter*_), where *P* and *D* denote numbers of protein monomers and dimers, is a decreasing function of *T*. In the case when *r*_*g*_=0 (no competition between the two genes), *R*_*protein*_ changes about three-fold from 2 for *T*=20 min to 0.64 for *T*=120 min, Fig. 3a. The sensitivity of the system to the doubling time significantly increases with gene competition strength. For high *r*_*g*_=0.0035, when the toggle exhibits bistability, *R*_*protein*_ changes more than 1500-fold from 32.4 for *T*=20 min to 0.02 for *T*=120 min, and even for a two-fold change of *T* (from 30 to 60 min), *R*_*protein*_ changes over 300-fold.

An increase of gene competition also increases the stability of the two toggle states. In the symmetric case (*T*=40 min) for *r*_*g*_=0.002 the state-to-state Mean First Passage Time (MFPT) is 25 h, while for *r*_*g*_=0.0035, MFPT = 58 h, Additional file 4: Figure S4. For *T*≠40 min the two states are not equiprobable and the MFPTs between them are different. For the latter *r*_*g*_ value, MFPTs are equal 118 and 10 h for *T*=30 min, and to 34 and 987 h for *T*=60 min. The high *r*_*g*_ values imply that *R*_*protein*_ is sensitive to changes of *T*, but also the time in which the toggle reaches new equilibrium is longer, Fig. 3c-f. Nevertheless, the change of *R*_*protein*_ 6 h after increase or decrease of *T* increases with *r*_*g*_ (in the considered *r*_*g*_ range), Fig. 3b. Within 6 h, for *r*_*g*_=0.0035, *R*_*protein*_ changes about two-fold when *T* changes from 40 to 60 or to 30 min.

**Fig. 4:**
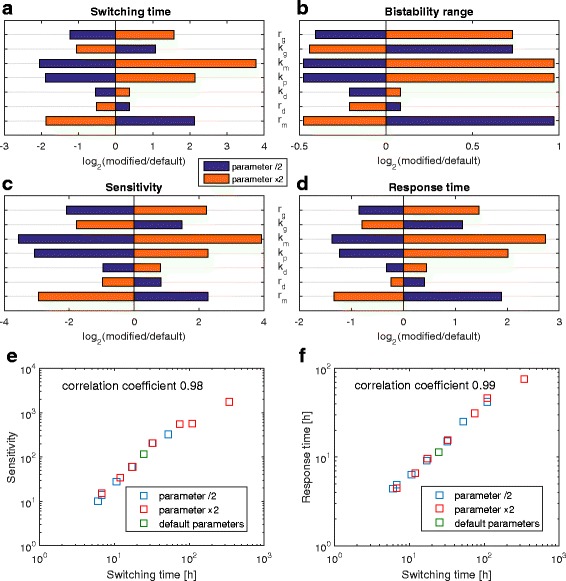
Parameter sensitivity analysis. Each of the model parameters was separately increased or decreased two-fold and the effect of these modifications on four model metrics was analyzed in (**a**) to (**d**). *r*_*g*_ was increased and decreased from the value of 0.002. **a** The toggle *switching time* was calculated for *T*=40 min, when the two state-to-state MFPT are equal. **b** The ratio of doubling times limiting the *bistability range* in the deterministic approximation (see “Methods” section, “Deterministic approximation” subsection). **c** Toggle *sensitivity* to the change of doubling time defined as the average protein ratio ((*P*_1_+2*D*_1_)/(*P*_2_+2*D*_2_)) for *T*=30 min divided by the average protein ratio for *T*=60 min. **d** *Response time* defined as the characteristic time needed to reach new asymptotic value of $\log _{2}((P_{1}+2D_{1})/(P_{2}+2D_{2}))$ after change of the doubling time. Response times shown in (**d**) and (**f**) are geometric averages of response times for change of doubling time from *T*=40 min (for which $\log _{2}((P_{1}+2D_{1})/(P_{2}+2D_{2}))=0$) to *T*=30 and *T*=60 min. **e** Correlation between the switching time and the toggle sensitivity. **f** Correlation between the switching time and the response time

We verify robustness of the presented results by performing system sensitivity analysis in which each of the model parameters is separately increased or decreased twofold with respect to its default value, Fig. 4. We compute the impact of these modifications on four key model metrics: switching time, bistability range in the deterministic approximation, toggle sensitivity to increase or decrease of *T*, toggle response time, i.e. characteristic time in which the toggle adjust to new *T*, Fig. 4a-d. We found that there is a high degree of correlation between these four measures. Importantly, the two last measures, i.e. these on which the doubling time indicator is based, nearly perfectly correlate with the switching time, Fig. 4e-f. This implies that all toggle parameters influence sensitivity and response time of the indicator only via the toggle switching time. The desired characteristic of the indicator can be thus achieved for various sets of the toggle parameters as a change of one parameter can be almost fully compensated by appropriate changes of other parameters.

In the preceding analysis we considered toggle switch in which the expression of the competing genes was equal for *T*=40 min. We showed that such a toggle is a sensitive indicator of a doubling time departure from the default value of 40 min. Now, we show that by adjusting the copy numbers of the competing genes, $G_{\overline {ori}}$ and $G_{\overline {ter}}$, and loci of these genes, we can design a toggle for which the average expression of both genes is equal for a given doubling time from the range of 20 to 120 min. Such a toggle can be used as a sensitive reporter of increase or decrease of the doubling time with respect to the desired value.

**Fig. 5:**
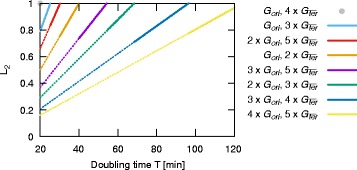
Genome loci of $G_{\overline {ter}}$ assuring equal average expression of both genes for a given doubling time. Distance, *L*_2_(*T*), of gene $G_{\overline {ter}}$ from the origin of replication assuring equal average expression of *G*_*ori*_ and $G_{\overline {ter}}$. For $G_{\overline {ter}}$ localized in *ter* *L*_2_=1. *L*_2_(*T*) dependence follows from the formula (1)

In Fig. 5 we considered 8 pairs of $G_{\overline {ori}}$ and $G_{\overline {ter}}$ copy numbers having 1-4 copies of $G_{\overline {ori}}$, and 2-5 copies of $G_{\overline {ter}}$. For the sake of simplicity we assume that $G_{\overline {ori}}$ is placed in the close vicinity of *ori* and we refer to this gene as *G*_*ori*_. Then for each pair of the gene copy numbers, from the formula (1), we calculate the position *L*_2_ of $G_{\overline {ter}}$ with respect to *ori*, as a function of *T*, for which the average expressions of the competing genes are equal. It follows from Eq. (1) that the toggle sensitivity to doubling time increases with the difference of distances of the competing genes to *ori* (*L*_2_−*L*_1_). Therefore, we are interested in the solutions in which distance *L*_2_ is the greatest. Such solutions are marked by the solid line in Fig. 5. For the considered pairs of gene copy numbers, *L*_2_>0.7. By increasing the maximal copy numbers of both genes, one can obtain an equilibrium for greater values of *L*_2_.

## Discussion

Our analysis indicates that a gene circuit based on two competing genes located far from each other on the chromosome, one near *ori* and the other near *ter*, would be sensitive to nutrient-dependent changes in bacterial doubling time. Temperature-dependent doubling time changes in *E. coli* do not influence the ratio of *ori* to *ter* copies [21], and as a result will not influence the designed system. CRISPR/CAS9 genome editing can be applied to place two competing, fluorescent protein-encoding genes and their promoters in desired loci of the bacterial genome [19]. To avoid the influence of local sequences, which can significantly alter the expression of the inserted genes [22], both competing genes should be isolated from neighboring sequences by an upstream and two downstream terminator sequences [20].

The proposed circuit can be based on any pair of repressors. The repressor genes should be preceded by promoters under the control of the opposing repressor. As shown in Fig. 5 for a given doubling time the balance between the competing genes can be achieved by inserting these genes in appropriate copy numbers. By modifying numbers of inserted gene copies one can also compensate for differences in strengths of gene promoters or half-lives of their transcripts. As shown in Fig. 5, the final tuning can be done by placing one of the genes not in the immediate vicinity of *ter* but at the appropriate distance from it. Bacteria containing such a circuit would report nutrient changes that influence its doubling time by changing color. Based on the performed sensitivity analysis one can expect that the doubling time indicator can work for a broad range of the toggle parameters. This is because a change of a given parameter can be almost fully compensated by appropriate changes of the remaining parameters. The two key measures of the doubling time indicator, i.e. its sensitivity to the doubling time and response time, nearly perfectly correlate with the toggle switching time, thus all parameters influence functioning of the indicator only via modulating the toggle switching time.

We have proposed possibly the simplest design of an indicator i.e. such that all parameters associated with expression of the competing genes are equal, while the balance between genes placed in different chromosomal positions is achieved by inserting higher number of copies of the gene inserted closer to *ter*. The ratio of the inserted gene copies and chromosomal positions of the genes determine the value of the doubling time for which their expected expression will be equal. In the simplest analyzed case the competing genes are placed respectively in the close vicinity of *ori* and close vicinity of *ter*, and there is one inserted copy of the gene placed near *ori* are two inserted copies of the gene placed near *ter*. In this case the perfect balance between the genes is achieved for the doubling time of 40 min for which the number of *ori* is twice higher than number of *ter*, which assures that the effective numbers of copies of both genes are equal. Alternatively to the presented design, the balance between the competing genes can be reached when both genes are inserted in the same number of copies, but the gene closer to *ori* is under a weaker promoter, its transcript is less stable or it has suboptimal ribosome-binding sites. Also in this design the doubling time would influence the ratio of the competing gene copies and thus the relative stability of the two toggle states. Importantly, this influence is strongest when the difference of distances of the competing genes to *ori* is the largest, i.e. when one gene is placed near *ori* and the other is placed near *ter*.

Our analysis sheds light on the potential influence of the doubling time on non-synthetic circuits in bacteria. In most cases the regulatory system dependence on the doubling time may be an unwanted property and bacteria typically reduce the sensitivity to the growth rate by grouping genes responsible for a given function in operons [23], which occupy small DNA segments and are transcribed into single mRNA chains. However, making some processes sensitive to the growth rate can be advantageous. In the case when the growth rate-sensitive pathway exhibits bistability, change of the doubling time can induce state-to-state transitions. Such a mechanism can be exploited by cell populations to differentiate into fast growing and dormant cells resistant to harsh conditions.

## Conclusions

In this study we propose how to design a synthetic bistable system that responds to change in the bacterial doubling time. We suggest that employing nutrient dependent variation in the ratio of *ori* to *ter* copies one can build a genetic toggle switch in which shortening of the cell cycle induces switching to a high expression level of the gene placed in the vicinity of *ori*, while decreasing growth rate leads to a transition to a high expression level of the gene placed in the vicinity of *ter*. Both sensitivity and response time of the proposed indicator increase and nearly perfectly correlate with the toggle switching time. Experimental validation of the proposed circuits could provide an insight into growth rate dependent changes in gene expression.

## Methods

In this section we present details of the designed model, performed stochastic simulations and analysis. Before we introduce details of the genetic toggle switch system, we explain the impact of cell growth and division on the considered system. First, we present the genome replication model. As we place the two genes of the toggle system in the vicinity of *ori* and *ter*, we focus on the variation of number of copies of *ori* and *ter* during the cell cycle for different growth rates. Next, after introducing the toggle switch model, we define the deterministic approximation of the system. This approximation is applied to analyze the systems bistability and its dependence on the doubling time and repression level of the competing genes. Finally, we define a Markov process describing the designed toggle switch in growing and dividing cells and explain how the state-to-state MFPTs were calculated.

### Model of genome replication, cell growth and division

We consider doubling times between 20 min and 120 min. Schematic of the genome replication progress for different doubling times *T* is shown in Additional file 1: Figure S1. For a doubling time of 20 min, at *t*=0 (i.e. at the division), the most advanced replication round, which begun two cycles earlier, terminates. At the same time the second replication round proceeds, and a new round starts. As a result, through the whole cell cycle there are eight copies of *ori* (violet circles), two copies of *ter* (green circles) and six pairs of active replication forks (gray triangles) are present. For different doubling times a number of timepoints are shown to illustrate subsequent steps of genome replication, each with different topology of DNA (i.e. different number of *ori* or *ter*).

The actual numbers of *ori* and *ter*, *N*_*ori*_(*T*,*t*) and *N*_*ter*_(*T*,*t*) (shown in the second column in Additional file 1: Figure S1), averaged over cell cycle, denoted respectively *S*_*ori*_(*T*) and *S*_*ter*_(*T*), can be calculated for different doubling times based on the genome replication scheme:

2 $$ S_{ori}(T)=\left\{ \begin{array}{ll} \frac{240}{T}-4 & T<30\:\text{min,}\\ \frac{120}{T} & 30\leq T<60\:\text{min,}\\ 1+\frac{60}{T} & T\geq 60\:\text{min;} \end{array}\right.  $$

3 $$ S_{ter}(T)=1+\frac{20}{T},  $$

where *T* is in minutes.

*S*_*ori*_(*T*) can be approximated (see Additional file 2: Figure S2a) by $S_{\textit {ori},appr}(T) = S_{ter}\cdot 2^{\frac {40}{T}}$, which follows from a more general formula [20]:

4 $$ S_{L_{i}}(T)=S_{ter}\cdot2^{(1-L_{i})\frac{40}{T}},  $$

describing approximate average gene copy number, where *L*_*i*_ is the distance from the position of the gene to the origin of replication (0≤*L*_*i*_≤1). *S*_*ori*,*appr*_(*T*) and *S*_*ter*_(*T*) serve to approximate copy numbers of the competing genes in the deterministic approximation of the model, see “Model of the genetic toggle switch” subsection.

The total rate of transcription in *E. coli* is limited by the concentration of free functional RNA polymerase rather than by the DNA concentration [5]. Based on the fact that the number of RNA polymerases transcribing mRNA is independent to the doubling time, we assume that the transcription rate *k*_*m*_ is inversely proportional to the DNA per cell content, which is denoted as *S*(*T*) and calculated based on the genome replication scheme as the gene copy number averaged over all loci and over the cell cycle, see Table 1. *S*(*T*) can be approximated by $S_{appr}(T) = \frac {1}{\ln (2)}\left (\frac {T}{40}+\frac {1}{2}\right)\left (2^{\frac {40}{T}}-1\right)$ resulting from the Eq. (4). To account for a constant protein concentration [5, 24] we assumed that the translation rate is proportional to the growth rate multiplied by the cell volume after division.

The cell volume after division, *V*_0_(*T*), decreases with increasing doubling time *T*, and for considered doubling times between 20 and 120 min can be expressed as: $V_{0}(T)=V_{0}(T=40\; min) \times \left (\frac {1}{3}(\frac {40}{T})^{2}+\frac {2}{3} \right)$. Such *V*_0_(*T*) is a fit for experimentally observed doubling time dependent changes in cell size [25, 26]. We will use units in which *V*_0_(*T*=40 *m* *i* *n*)=1. For a given doubling time, the cell volume growth is exponential, $V(t,T)=V_{0}(T)e^{\ln (2)\frac {t}{T}}$. When the volume doubles, the cell divides. We assume that after division daughter-cells are equal in volume while mRNAs, proteins and protein dimers, are distributed between the daughter-cells following the binomial distribution with parameter 0.5, i.e. each molecule has equal probability to enter each of the daughter-cells. All bimolecular reaction rates in the model are inversely proportional to the cell volume.

### Model of the genetic toggle switch

For most of the analysis we focus on the case in which there is a single copy of the gene *G*_*ori*_ inserted to the genome in the vicinity of *ori*, and two copies of the gene *G*_*ter*_ inserted in the vicinity of *ter*. As shown in Additional file 1: Figure S1 the number of *ori* remains twice higher than the number of *ter* through whole cell cycle when the doubling time equals 40 min. Therefore, the above assumption assures that the actual numbers of *G*_*ori*_ and *G*_*ter*_ copies are equal for doubling time of 40 min, which implies equal average expression of the two competing genes. Each gene copy can be repressed by binding of a competing protein dimer or activated when such a dimer is released. mRNA is transcribed with equal rates from all active gene copies. The model includes the following processes: DNA replication, gene repression and activation, mRNA synthesis and degradation, protein translation and dimerization, cell volume growth and division. As a typical protein half-life in bacteria is much longer than the division time, we assume that the protein number decreases only due to cell division. The corresponding reaction rates are listed in Table 1.

Additionally we analyzed a variant of the system in which different copy numbers of the competing genes were inserted into the genome, see Fig. 5. We considered eight different ratios, *R*, of inserted copy numbers of the two genes: 1/4, 1/3, 2/5, 1/2, 3/5, 2/3, 3/4, 4/5. We assume that the gene inserted in the smaller number of copies is placed in the vicinity of *ori*, and calculate the position, *L*_2_(*T*,*R*), of gene $G_{\overline {ter}}$ assuring equal average expression of competing genes for a given doubling time *T*. $L_{2}(T,R)=-\frac {T}{C}\log _{2}R$ was calculated using the formula (4), where *C*=40 min is the genome replication time.

#### Deterministic approximation

The deterministic approximation is coarse for bacteria, for which characteristic numbers of molecules are small, but can be employed to identify system bistability and serve as a reference for stochastic simulations.

In the deterministic variant of the model we approximate the copy numbers of gene *G*_*ori*_ and *G*_*ter*_, by the average numbers of these genes copies over the cell cycle. Gene states and molecule levels are denoted with the same symbols as gene names and molecules names. In this model variant, continuous variables *G*_*ori*_ and *G*_*ter*_ denote gene activities, while continuous variables *M*_*ori*_, *M*_*ter*_, *P*_*ori*_, *P*_*ter*_, *D*_*ori*_, *D*_*ter*_ denote the levels of mRNAs, protein monomers, and protein dimers. Gene activities, *G*_*ori*_ and *G*_*ter*_∈[0 1], are regulated by competing dimers binding and dissociation, and the transcription from a given gene is proportional to the number of its copies. We consider implicit cell division, i.e. the dilution terms proportional to $\frac {\ln (2)}{T}$ are included in the equations. The cell volume is assumed to be constant for a given doubling time *T* and equal 2*V*_0_(*T*), i.e. equal to the cell volume just before division. The dynamics of the system is described by the following ordinary differential equations system:

$$\begin{array}{@{}rcl@{}} \frac{d G_{ori}}{d t} &=& k_{g}(1-G_{ori})-r_{g}(t,T)D_{ter}G_{ori}, \\ \frac{d G_{ter}}{d t} &=& k_{g}(1-G_{ter})-r_{g}(t,T)D_{ori}G_{ter}, \\ \frac{d M_{ori}}{d t} &=& S_{ori}(T) k_{m}(T) G_{ori}-\left(r_{m}+\frac{\ln(2)}{T}\right)M_{ori}, \\ \frac{d M_{ter}}{d t} &=& 2 S_{ter}(T) k_{m}(T) G_{ter}-\left(r_{m}+\frac{\ln(2)}{T}\right)M_{ter}, \\ \frac{d P_{i}}{d t} &=& k_{p}(T) M_{i}-\frac{\ln(2)}{T}P_{i}+2\left(r_{d} D_{i} -\frac{1}{2}k_{d}(t,T) P_{i}^{2}\right), \\ \frac{d D_{i}}{d t} &=& \frac{1}{2}k_{d}(t,T) P_{i}^{2}- \left(r_{d}+\frac{\ln(2)}{T}\right)D_{i}, \end{array} $$

where *i* denotes either *ori* or *ter*. The reaction rate constants *k*_*g*_, *k*_*m*_, *k*_*p*_, *k*_*d*_, *r*_*g*_, *r*_*m*_ and *r*_*d*_ are listed in Table 1.

In Additional file 3: Figure S3 we show stable stationary states of protein monomer levels as a function of doubling time *T*. These stationary states were obtained by slowly increasing (or decreasing) doubling time *T*. The solid lines denote stable stationary states for doubling time increasing from 20 to 120 min, while the dashed lines denote stable stationary states for decreasing doubling time from 120 to 20 min. For *r*_*g*_=0.002 and *r*_*g*_=0.0035 we observe a hysteretic response to doubling time change implying bistability in the system.

#### Stochastic model and simulations

The stochastic simulations of the system were performed using the Gillespie-type algorithm [27] implemented in C++. The only modification to the original Gillespie algorythm is that the cell volume (influencing bimolecular reactions) is updated after each time step. As the volume changes slowly compared to the fastest reaction, such approach is justified [16, 28]. The example stochastic trajectories for three doubling times 30, 40, and 60 min are provided in Additional file 6: Figure S6. The computational code is provided in Additional file 7: Computational Code.

The stochastic model of the system is defined by the time continuous Markov process. In this model variant states of *N*_*ori*_(*T*,*t*) copies of gene *G*_*ori*_ denoted by *G*_*ori*,*j*_ (with 1≤*j*≤*N*_*ori*_(*T*,*t*)) are discrete random variables and assume values 0 for a repressed gene copy and 1 for an active gene copy. States of 2*N*_*ter*_(*T*,*t*) copies of gene *G*_*ter*_ denoted by *G*_*ter*,*k*_ (with 1≤*k*≤2*N*_*ter*_(*T*,*t*)) are defined analogously. As in the deterministic approximation, the molecule numbers are denoted with the same symbols as their names. *M*_*ori*_, *M*_*ter*_, *P*_*ori*_, *P*_*ter*_, *D*_*ori*_, $D_{ter} \in \mathbb {N}$ denote thus random variables, respectively numbers of mRNA, protein monomers and dimers (Fig. 1). Each of the copies of gene *G*_*ori*_ can be repressed by a dimer *D*_*ter*_, while a dimer *D*_*ori*_ can repress any of the gene *G*_*ter*_ copies. DNA replication causes dissociation of DNA-bound proteins and accordingly, we assume that when each of the genes is replicated, its repressor molecule dissociates from DNA rendering the two arising gene copies active. The processes included in the model and their propensities are listed below:

$${{\begin{aligned} &(G_{ori,j}\,=\,0,\!D_{ter}\,=\,d_{ter})\!\rightarrow \!(G_{ori,j}\,=\,1,\!D_{ter}\,=\,d_{ter}+1) \qquad\! k_{g}(1\,-\,G_{ori,j}),\\ &(G_{ori,j}\,=\,1,\!D_{ter}\,=\,d_{ter})\!\rightarrow\! (G_{ori,j}\,=\,0,D_{ter}\,=\,d_{ter}-1) \qquad\!\! r_{g}(t,T)d_{ter} G_{ori,j},\\ &(G_{ter,k}\,=\,0,D_{ori}\,=\,d_{ori})\!\rightarrow\! (G_{ter,k}\,=\,1,D_{ori}\,=\,d_{ori}+1) \quad\,\,\, k_{g}(1\,-\,G_{ter,k}),\\ &(G_{ter,k}\,=\,1,D_{ori}\,=\,d_{ori})\!\rightarrow\! (G_{ter,k}\,=\,0,\!D_{ori}\,=\,d_{ori}-1) \quad\,\,\,\, r_{g}(t,T)d_{ori} G_{ter,k},\\ &M_{ori}\,=\,m_{ori}\!\rightarrow\! M_{ori}\,=\,m_{ori}\,+\,1 \qquad\qquad\qquad\qquad\,\,\,\! k_{m}(T)\Sigma_{j=1}^{N_{ori}(T,t)} G_{ori,j},\\ &M_{ter}\!=vm_{ter}\!\rightarrow\! M_{ter}\,=\,m_{ter}\,+\,1 \qquad\qquad\qquad\qquad\!\! k_{m}(T)\Sigma_{k=1}^{2N_{ter}(T,t)}\! G_{ter,k},\\ &M_{i}\,=\,m_{i}\!\rightarrow\! M_{i}\,=\,m_{i}\,-\,1 \qquad\qquad\qquad\qquad\qquad\, r_{m} m_{i},\\ &P_{i}\,=\,p_{i}\!\rightarrow \!P_{i}\,=\,p_{i}\,+\,1 \qquad\qquad\qquad\qquad\qquad\quad\, k_{p}(T) m_{i},\\ &(P_{i}\,=\,p_{i},D_{i}\,=\,d_{i})\!\rightarrow\! (P_{i}\,=\,p_{i}\,-\,1,\!D_{i}\,=\,d_{i}\,+\,1) \,\, \qquad\qquad k_{d}(t,T)p_{i} (p_{i} -1),\\ &(D_{i}\,=\,d_{i},P_{i}\,=\,p_{i})\!\rightarrow\! (D_{i}\,=\,d_{i}\,-\,1,\!P_{i}\,=\,p_{i}\,+\,1) \qquad\qquad\,\, r_{d} d_{i}, \end{aligned}}} $$

where subscript *i* denotes either *ori* or *ter*, 1≤*j*≤*N*_*ori*_(*T*,*t*) and 1≤*k*≤2*N*_*ter*_(*T*,*t*). The reaction rate constants are listed in Table 1.

In the case when the deterministic approximation of the system exhibits bistability (see Additional file 3: Figure S3) we calculated the mean transition times between the two states of the toggle switch. We assume that the transition arises when the numbers of protein monomers and of protein dimers associated with the initially repressed gene becomes larger than these of the initially dominating gene. It is expected that state-to-state switching times follow the exponential distribution [29]. In fact, based on 10^5^ transitions we found that an exponential function perfectly fits histograms of transition times, except for the shortest transition times arising from fluctuations around the unstable stationary state with low expression of both competing genes. Therefore, we determine the state-to-state MFPT, not by averaging over transition times, but by fitting *a* *e* ^−*λx*^ to the truncated histograms. As shown in Additional file 4: Figure S4, 1/*λ* depends on the truncation time, but stabilizes as this time increases. We thus define switching time as an asymptotic value of 1/*λ*.

## Additional files

Additional file 1 **Figure S1.** Genome replication model. (PDF 54 kb)

Additional file 2 **Figure S2.** Gene copy numbers as a function of the doubling time. (PDF 77 kb)

Additional file 3 **Figure S3.** Stable stationary states of protein monomers level for different doubling times and different repression levels. (PDF 97 kb)

Additional file 4 **Figure S4.** MFPT between the two states of the toggle switch. (PDF 97 kb)

Additional file 5 **Figure S5.** Stationary distributions of protein level based on stochastic simulations for different doubling times and different repression levels. (PDF 59 kb)

Additional file 6 **Figure S6.** Sample stochastic trajectories for three doubling times: 30, 40, and 60 min. (PDF 161 kb)

Additional file 7 Computational Code. C++ computational code. (CPP 46 kb)

## Abbreviations

MFPT: Mean first passage time
*ori*: Origin of replication
*ter*: Terminus of replication
