# Supplementary material for: Genetic toggle switch controlled by bacterial growth rate
Source: BMC Syst Biol. 2017 Dec 2;11:117. doi: 10.1186/s12918-017-0483-4 (PMC5712128; doi:10.1186/s12918-017-0483-4)
Supplement: Supplementary file 1 — Figure S1. Genome replication model. (PDF 54 kb) [file 12918_2017_483_MOESM1_ESM.pdf]

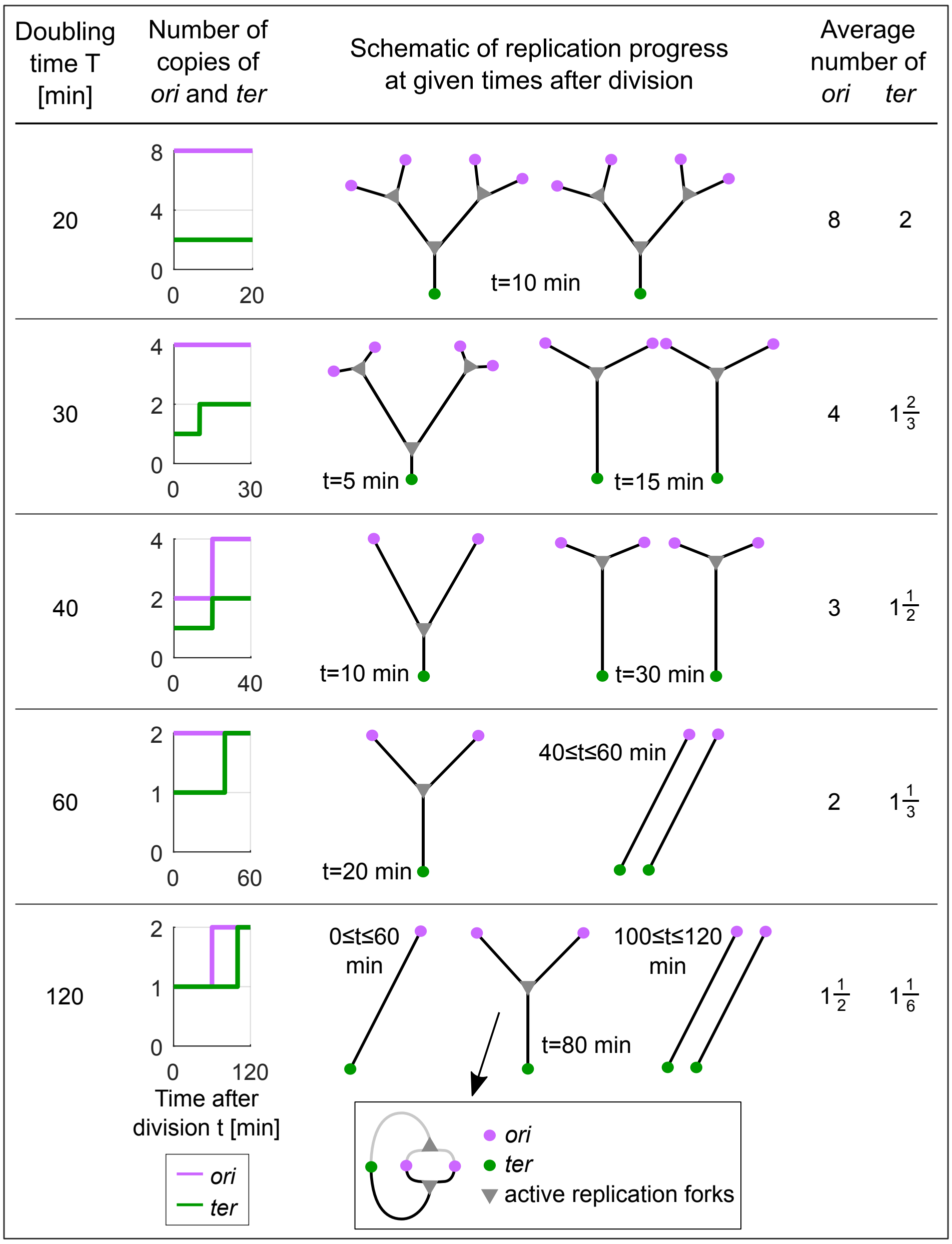

Figure S1: Genome replication based on the Cooper and Helmstetters's model [1, 3]. In the schematic of replication progress for simplicity only one half of a circular genome spanning *ori* and *ter* is shown (shown in black in the legend box).
