# Supplementary material for: Genetic toggle switch controlled by bacterial growth rate
Source: BMC Syst Biol. 2017 Dec 2;11:117. doi: 10.1186/s12918-017-0483-4 (PMC5712128; doi:10.1186/s12918-017-0483-4)
Supplement: Supplementary file 2 — Figure S2. Gene copy numbers as a function of the doubling time. (PDF 77 kb) [file 12918_2017_483_MOESM2_ESM.pdf]

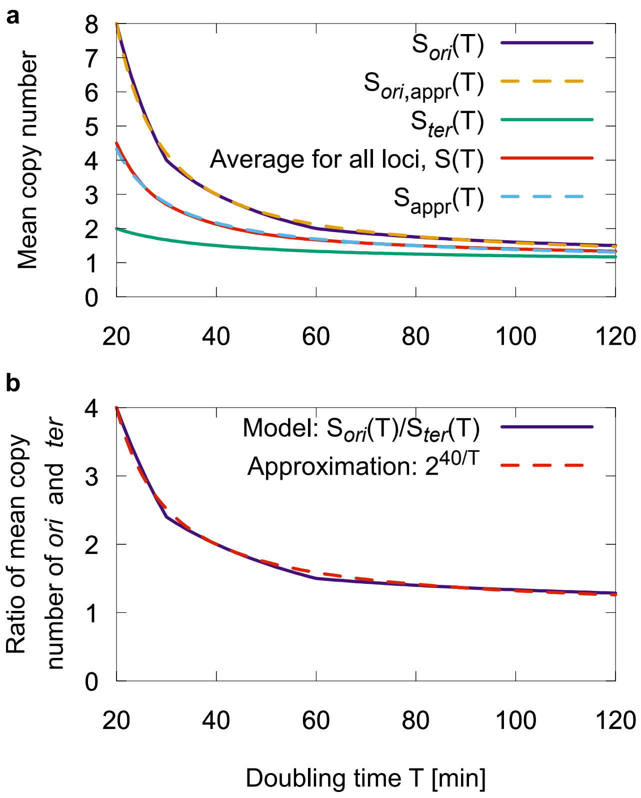

Figure S2: Gene copy numbers as a function of the doubling time. (a) Copy numbers of *ori* and *ter* averaged over the cycle, $S_{ori}(T)$ and $S_{ter}(T)$, and the number of gene copies averaged over all genome loci and over cell cycle, $S(T)$, as a function of doubling time T. Approximate values $S_{ori,appr}$ and S_{appr} (dashed lines) are calculated based on S_{ter} and the formula (4) describing approximate expression of a gene localized in L_i. (b) Ratio of mean copy number of *ori* and *ter* in the model compared to approximated ratio based on the formula (4)
