# Supplementary material for: Genetic toggle switch controlled by bacterial growth rate
Source: BMC Syst Biol. 2017 Dec 2;11:117. doi: 10.1186/s12918-017-0483-4 (PMC5712128; doi:10.1186/s12918-017-0483-4)
Supplement: Supplementary file 3 — Figure S3. Stable stationary states of protein monomers level for different doubling times and different repression levels. (PDF 97 kb) [file 12918_2017_483_MOESM3_ESM.pdf]

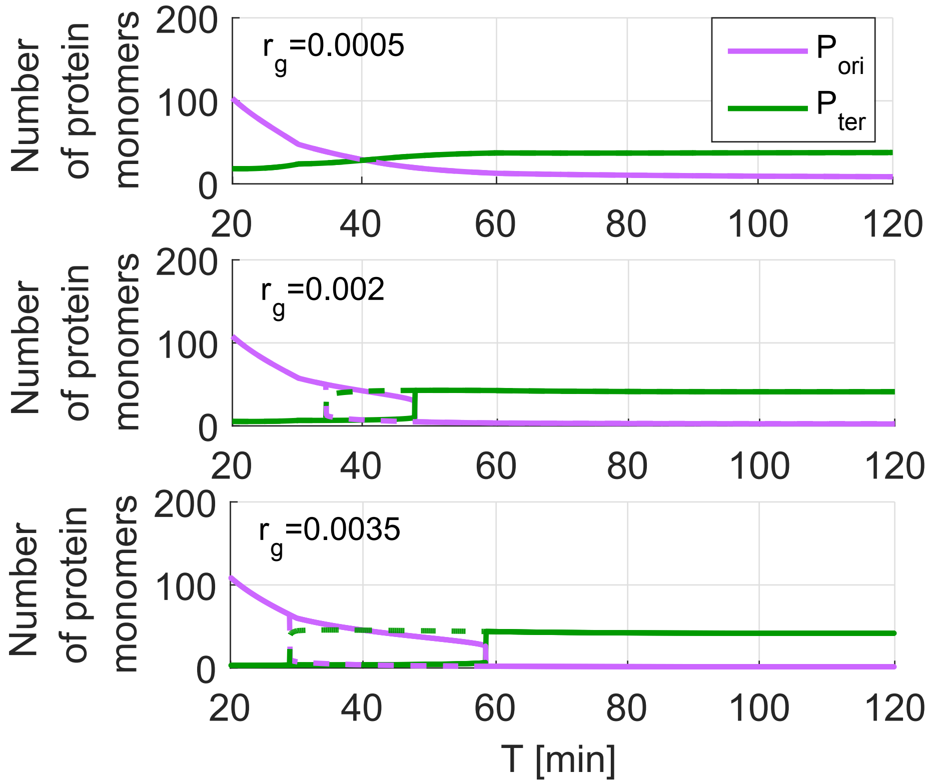

Figure S3: Stable stationary states of protein monomers level for different doubling times T and different repression levels r_g. Bistability range in the deterministic approximation of the model increases with the increasing inhibition rate r_g. For low level of repression, $r_g = 0.0005$, the system is monostable for all considered doubling times T. For stronger repression, the bistability arises first for doubling time $T = 40$ min, and then the range of doubling times for which the system is bistable increases with repression level.
