# Supplementary material for: Genetic toggle switch controlled by bacterial growth rate
Source: BMC Syst Biol. 2017 Dec 2;11:117. doi: 10.1186/s12918-017-0483-4 (PMC5712128; doi:10.1186/s12918-017-0483-4)
Supplement: Supplementary file 4 — Figure S4. MFPT between the two states of the toggle switch. (PDF 97 kb) [file 12918_2017_483_MOESM4_ESM.pdf]

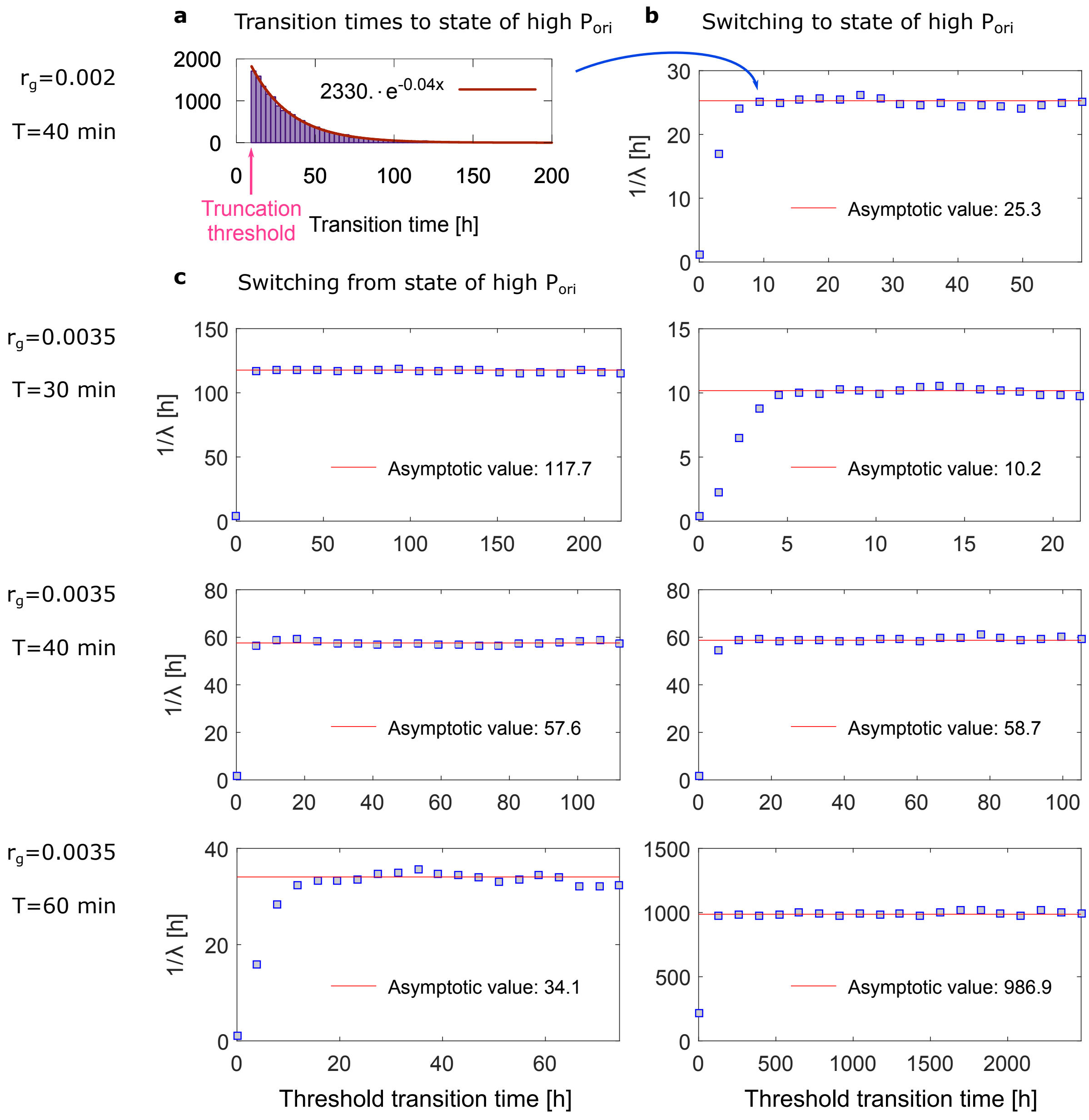

Figure S4: Mean switching times between the two states of the toggle switch. (a) Truncated histogram of transition times between two states and its exponential fit $a \cdot e^{-\lambda}$ (red). (b-c) Mean switching times between the two states of the toggle switch (marked red) are calculated as an asymptotic value of $1/\lambda$.
