# Supplementary material for: Genetic toggle switch controlled by bacterial growth rate
Source: BMC Syst Biol. 2017 Dec 2;11:117. doi: 10.1186/s12918-017-0483-4 (PMC5712128; doi:10.1186/s12918-017-0483-4)
Supplement: Supplementary file 5 — Figure S5. Stationary distributions of protein level based on stochastic simulations for different doubling times and different repression levels. (PDF 59 kb) [file 12918_2017_483_MOESM5_ESM.pdf]

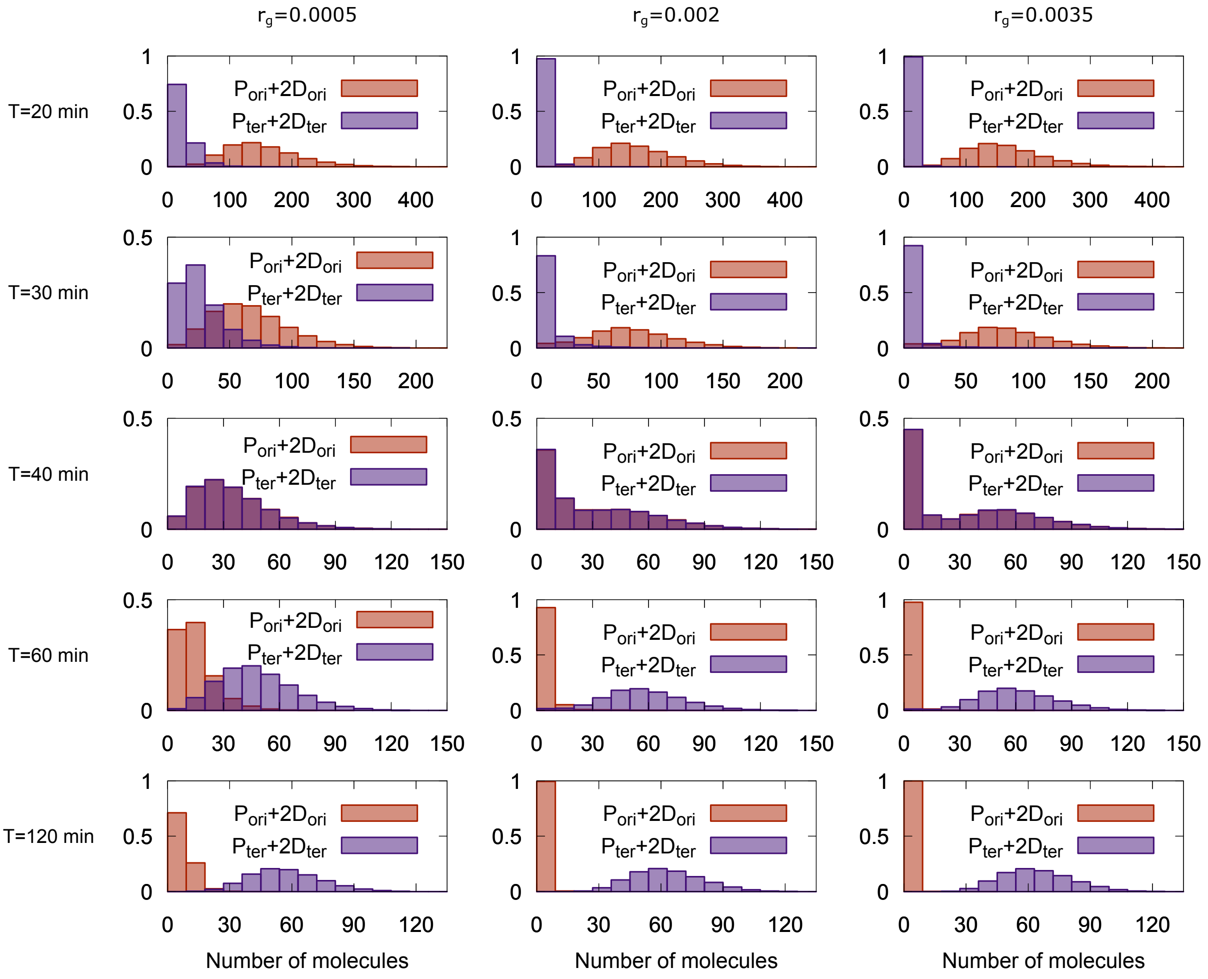

Figure S5: Stationary distributions of protein level based on stochastic simulations for different doubling times T and different levels of repression r_g.
