# Supplementary material for: Genetic toggle switch controlled by bacterial growth rate
Source: BMC Syst Biol. 2017 Dec 2;11:117. doi: 10.1186/s12918-017-0483-4 (PMC5712128; doi:10.1186/s12918-017-0483-4)
Supplement: Supplementary file 6 — Figure S6. Sample stochastic trajectories for three doubling times: 30, 40, and 60 min. (PDF 161 kb) [file 12918_2017_483_MOESM6_ESM.pdf]

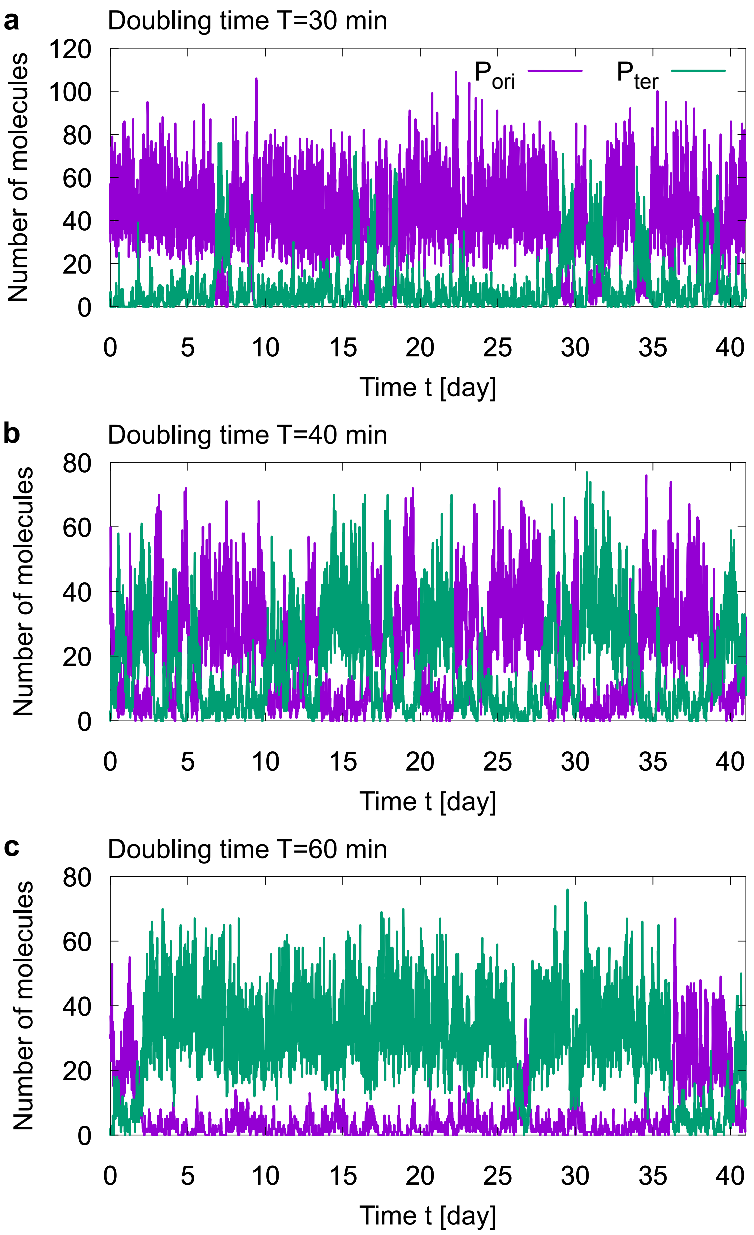

Figure S6: Number of protein monomers from sample stochastic trajectories computed for $r_g = 0.002$ and for three doubling times: 30, 40, and 60 min.
